# An Investigation on the Most Likely Failure Locations in the BEoL Stack of a 20 nm Chip Due to Chip Package Interaction with the Use of Novel Semi-Elliptical Cracks

**DOI:** 10.3390/mi14101953

**Published:** 2023-10-19

**Authors:** Ganglong Li, Yidian Shi, Andrew A. O. Tay, Zhilin Long

**Affiliations:** 1 School of Civil Engineering and Mechanics, Xiangtan University, Xiangtan 411105, China; 2 School of Mechanical and Electronic Engineering, East China University of Technology, Nanchang 330013, China; 3 School of Mechanical and Electrical Engineering, Central South University, Changsha 410083, China; sydian@csu.edu.cn; 4 Division of Engineering Product Development, Singapore University of Technology and Design, Singapore 487372, Singapore

**Keywords:** reliability, chip-package interaction, delamination, thermal stress, competitive failure

## Abstract

The era of 20 nm integrated circuits has arrived. There exist abundant heterogeneous micro/nano structures, with thicknesses ranging from hundreds of nanometers to sub-microns in the IC back end of the line stack, which put stringent demands on the reliability of the device. In this paper, the reliability issues of a 20 nm chip due to chip–package interaction during the reflow process is studied. A representative volume element of the detailed complex BEoL structure has been analyzed to obtain mechanical properties of the BEoL stack by adopting a sub-model analysis. For the first time, semi-elliptical cracks were used in conjunction with J-integral techniques to analyze the failure caused by Chip-to-Package Interaction for a 20 nm chip. The Energy Release Rate(ERR)for cracks at various interfaces and locations in the BEoL stack were calculated to predict the most likely mode and location of failure. We found that the ERR of interfacial cracks at the bottom surface of the interconnects are, on average, more than double those at the sidewalls, which are in turn more than double the number of cracks in the low-*k* inter-layer dielectric. A total of 500 cycles of thermal shock were conducted, which verified the predictions of the finite element simulations.

## 1. Introduction

As the feature size of modern integrated circuits (IC) continues to decrease, the width of the interconnects and the inter-layer dielectrics (ILD) progressively scale down accordingly [1,2,3,4]. In order to reduce the RC delay aggravated by the downsizing trend, materials with low dielectric constant, commonly known as “low-*k*”, are widely used as dielectrics in the back end of line (BEoL) stack [5]. However, the semiconductor chip generally becomes mechanically weaker due to the introduction of low-*k* dielectrics, while the package stress increases in many cases, posing increasing CPI challenges. The semiconductor industry has spent tremendous effort to develop and integrate low-*k* and ultra-low-*k* (ULK) materials into the backend of the line (BEoL) stack for the clear benefits of faster performance and lower power consumption. However, low dielectric constants directly correlate to weaker chemical bonding in the materials, which inherently reduces mechanical strength [6]. Such reliability issues are receiving increasing attention from IC manufacturing designers [7,8].

In recent years, with the rise of advanced packaging schemes, the chip–package interaction (CPI) has become an issue of particular concern, due to its effect on the reliability of the final products [9]. Due to the difficulties in measuring the stress and strain in the BEoL stack, most attempts to define this stress have been through finite element simulation. However, the huge difference in dimensions between the interconnects in the ILDs and the package makes it unrealistic to calculate the stress distribution in the entire package, as well as in the detailed structures, using one finite element model. Various methods have been proposed by researchers to determine the stress distribution in the on-chip BEoL. Mercado et al. [10,11] used a 4-level sub-model method to calculate the stress in the low-*k*/passivation layer undergoing the packaging process and showed that the multi-level sub-model technique can be used to evaluate the stress in the BEoL stack at a much smaller scale than in the package. The main failure mode of the device was found to be delamination, and the packaging process directly affects the failure. Zhang et al. [12] used a multi-level, finite-element sub-model and thermal deformation tests to investigate the warpage of a 45 nm chip during the annealing process, as well as the reliability of the BEoL layer due to CPI. Liu et al. [13] used a three-level, finite-element sub-model to analyze the reliability issues caused by different dielectric materials in the BEoL layer and the presence of cracks in the dielectric material. They found that the use of certain crack-stop structures and appropriate underfill materials could effectively prevent delamination resulting from CPI.

The above works have some limitations in accuracy owing to the following factors: (i) the use of too many levels of sub-models, and (ii) the use of 2D models instead of more realistic 3D models to model the CPI issue, which is closely related to the structural complexity and requires special fineness. Some research has aimed to improve computational accuracy by adopting a 3D model with fewer levels of sub-modeling. For example, Wang et al. [14,15,16] used four-point bending tests to obtain the fracture toughness of the Cu/low-*k* interface and used the modified virtual crack closure technique (MVCCT) in a 3D model to analyze the fracture of the BEoL stack. Gao et al. [17] used experimental methods to study the failure of a 6-layer BEoL stack due to high temperature and moisture. They demonstrated that the capacitance of the device rose significantly, and failure occurred in the BEoL layer when it was subjected to a temperature higher than 400 °C in a high-humidity environment. Chihiro et al. [18] studied the failure of Cu/low-*k* interconnect structures with a feature size of 65 nm using the MVCCT in fracture mechanical to calculate the crack Energy Release Rate (ERR) for different underfill materials. Their results show that lead-free solder joints have the greatest impact on BEoL reliability due to CPI. Abhishek Tambat et al. [19] used classical elasticity theory to analyze chip failure from the perspective of crack initiation, substrate geometry, and solder material. They found that the cooling process, from a high temperature to room temperature, posed the greatest risk of failure and that using low-*k* and SiO_2_ materials instead of ultra-low-*k* materials significantly reduced the risk of CPI-induced failure.

In this paper, in order to improve the computational accuracy while maintaining efficiency, a two-level, finite-element sub-model was employed to minimize the computational errors due to excessive levels of sub-modeling. The equivalent mechanical properties of the complex structure of the BEoL stack were first obtained by using a representative volumetric element (RVE) and were subsequently used in 3D finite element simulations of a 20 nm chip package. The global-local, two-level sub-model was employed for the analysis of stress developed in the BEoL stack during a solder reflow process. Afterward, the ERR was calculated for virtual cracks at various locations in the BEoL stack in order to determine the most likely failure locations and failure modes. Furthermore, a more realistic elliptical crack front was employed, which is a significant improvement over other works to date. Lastly, an experiment involving 500 cycles of thermal shock was performed to validate the simulation results.

## 2. Modeling of BEoL Stack

### 2.1. Details of the BEoL Stack of the 20 nm Chip

**Figure 1:**
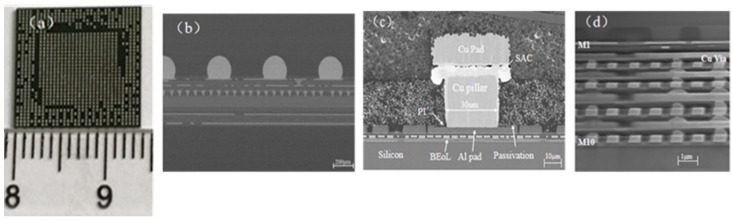
(**a**) Size of device. (**b**) Cross-sectional view of the whole package. (**c**) Cross-sectional SEM image of copper pillar interconnection. (**d**) Cross-sectional SEM image of BEoL.

As shown in Figure 1a, the overall size of the packaged 20 nm chip is 13.8 mm × 13.8 mm × 1.02 mm, and the number of input/output pins (I/O) is approximately 920. The chip was cut using a focused ion beam (FIB), and the cross-sectional details of the functional strata were obtained using SEM. As shown in Figure 1b, the chip is connected to the substrate by the Ball Grid Array Package. Details of the various layers of materials between the chip and the substrate is shown in the cross-section in Figure 1c. The microbump diameter of the copper pillar 30 μm, and the size of the opening in the PI layer is 20 μm. As can be seen from Figure 1d, the BEoL stack of the chip has a total of 10 metal layers, which are defined from metal layer 1 (M1) to metal layer 10 (M10), from top to bottom. Different metal layers are interconnected by Cu vias and filled with low-*k* dielectric material.

### 2.2. “Global-Local”Finite-Element Models

**Figure 2:**
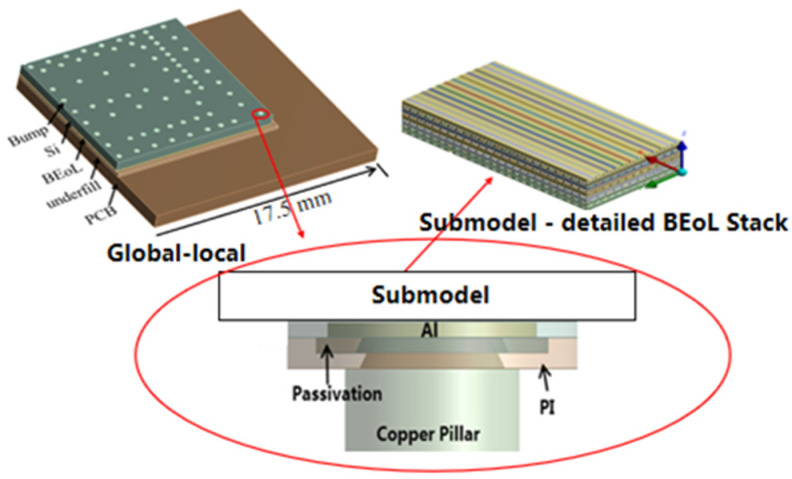
“Global-local” multi-level sub-modeling of test vehicle.

The test vehicle used in this study consists of the above-described chip bonded onto a substrate board, as shown in Figure 2. In order to obtain the thermal stress distribution with high precision and computational efficiency, the equivalent mechanical properties of the BEoL stack are first obtained and subsequently used in the “global-local” modeling to calculate the thermal stress distribution in the BEoL stack during a reflow process. As shown in Figure 2, the finite element model of the test vehicle is composed of PCB, underfill, BEoL, bumps, and chip; the bumps are composed of Cu pad, solder, Cu pillar, PI layer, passivation layer, and aluminum pad. The dimensions of the various components of the package are given in Table 1.

**Figure 3:**
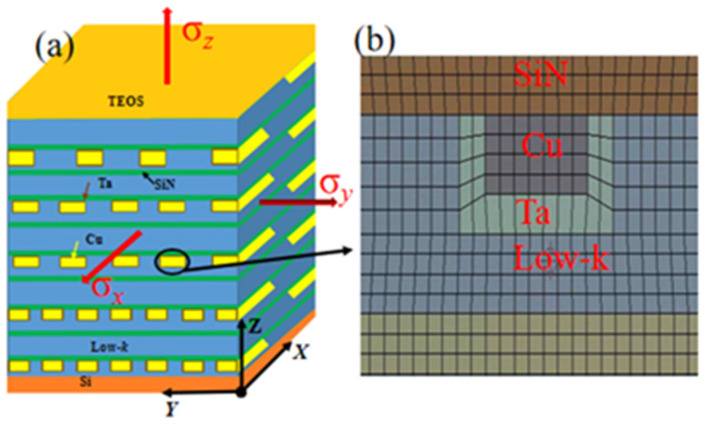
(**a**) Schematic diagram of the BEoL structure. (**b**) FE mesh around Cu interconnect showing Ta barrier layer and SiN etch stop.

As shown in Figure 3, multiple partitioning techniques have been used to ensure that all the finite elements used in modeling the structures of the device and package consist of only hexahedral elements for better computational accuracy.

#### 2.2.1. Effective Mechanical Properties of the BEoL Stack

In order to accurately calculate the stress distribution in the global model, the complex BEoL structure should be taken into account. This was executed by obtaining the effective mechanical properties of an RVE, which correspond to the typical BEoL structure, using finite element (FE) simulations. Such an RVE is illustrated in Figure 3a. The RVE of 4 × 4 × 8.2 μm^3^ in dimensions has a 10-layer Cu interconnect structure (Figure 1d), and the interconnect metal-layer is packed with a porous dielectric material. The Ta layer, as the diffusion barrier for Cu, and the SiN layer, as an etch stop, were both modeled, as shown in Figure 3b. The vertical Cu vias which provide signal transfer vertically across metal layers were assumed to be a transversely isotropic material. A uniform axial stress, x, was firstly applied to the RVE in the x direction, and the corresponding elongation, ΔL, was obtained using finite element analysis (FEA). The effective elastic modulus, Ex, and position ratio μ_yz_ in the x direction, μ_yz_, were then obtained through Equations (1) and (2), respectively:

(1) $$E_{x}=\frac{\sigma_{x}L}{∆L}$$

(2) $$\mu_{yz}=-\frac{L}{2∆L}\left(\frac{∆W}{W}+\frac{∆H}{H}\right)$$

where ΔW and ΔH are the extensions in the width and height directions. Similarly, axial stresses σ_y_ and σ_z_ are applied in the y and z directions, in turn, to obtain the effective elastic module and Poisson ratios in the y and z directions. The mechanical properties of the materials used in the finite element simulations are given in Table 2.

#### 2.2.2. Effect of the Width of the Cu Interconnects and the Number of Layers of BEoL Stack on the Equivalent Elastic Modulus

The effects of the number of layers in the BEoL stack and the width of the Cu interconnects, on the out-of-plane elastic modulus (E_z_) and the in-plane elastic modulus (E_x_, E_y_) of the BEoL stack, were investigated by performing the above mentioned FEA on the RVE, with varying heights determined by the number of BEoL layers. For this investigation, the thickness of the Ta barrier layer and the pitch of the copper interconnects are fixed at 0.1 μm and 1.6 μm, respectively. The mechanical properties and the dimensions of the components in the BEoL stack are shown in Table 2 and Table 3, respectively.

**Figure 4:**
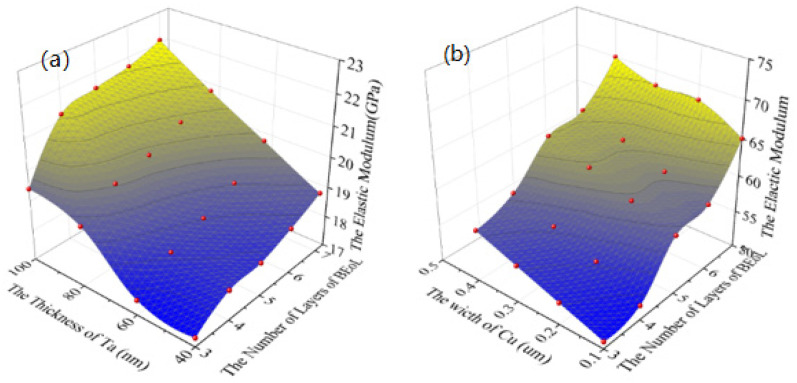
The relationship between equivalent elastic modulus and the width of Cu interconnects and the number of layers of BEoL: (**a**) Out-of-plane elastic modulus. (**b**) In-plane elastic modulus.

The results are plotted in Figure 4. The red points are the results of the FEA which are fitted by surfaces. The out-of-plane elastic modulus of the BEoL stack increases as the Cu width and the number of layers increase. This is mainly because the elastic modulus of the interconnect material, Cu (121 GPa), is much higher than the elastic modulus of the low-*k* dielectric (10.6 GPa), so increasing the width of the Cu directly leads to an increase in the out-of-plane equivalent elastic modulus. Similarly, increasing the number of layers in the BEoL structure increases the volume fraction of Cu in the BEoL structure, resulting in an increase in the out-of-plane elastic modulus. As can be seen in Figure 4b, the in-plane elastic modulus also shows the same trends.

The results are plotted in Figure 4. The red points are the results of the FEA which are fitted by surfaces. The out-of-plane elastic modulus of the BEoL stack increases as the Cu width and the number of layers increase. This is mainly because the elastic modulus of the interconnect material, Cu (121 GPa), is much higher than the elastic modulus of the low-*k* dielectric (10.6 GPa); therefore, increasing the width of the Cu directly leads to an increase in the out-of-plane equivalent elastic modulus. Similarly, increasing the number of layers in the BEoL structure increases the volume fraction of Cu in the BEoL structure, resulting in an increase in the out-of-plane elastic modulus.

**Figure 5:**
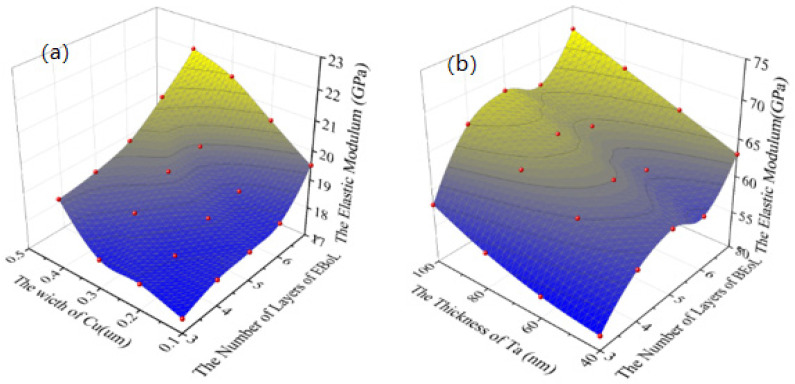
Variation of equivalent elastic modulus with a different number of layers and thickness of Ta barrier layer: (**a**) Out-of-plane. (**b**) In-plane.

Figure 5 shows that, with the width of Cu fixed at 0.4 μm, the equivalent out-of-plane elastic modulus increases with either the increase in Ta thickness or the increase in the number of layers, mainly due to an increase in the volume ratio of Ta in the BEoL structure with the increase in the thickness of the Ta layer.

#### 2.2.3. Thermal Stress in BEoL Stack during Solder Reflow

**Figure 6:**
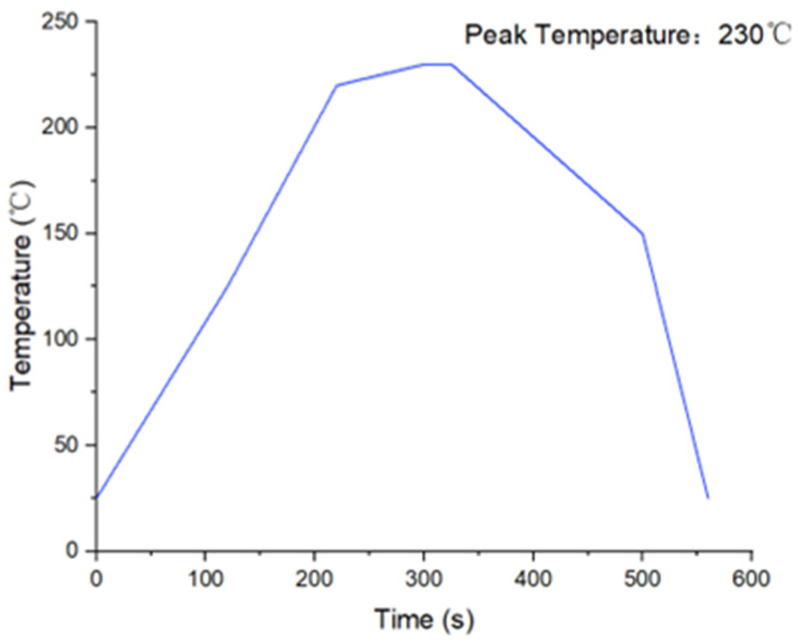
A typical reflow profile.

A mismatch of the coefficient of thermal expansion (CTE) of the constituent materials in the chip package causes large stresses during processes of large temperature variation, e.g., the solder reflow process, and the most simplified criterion for reliability assessment is the comparison of thermal stress versus the strength of the materials. As shown in Figure 6 in the simulation for solder reflow process, which specifies that the temperature should rise from 25 °C to 260 °C.

**Figure 7:**
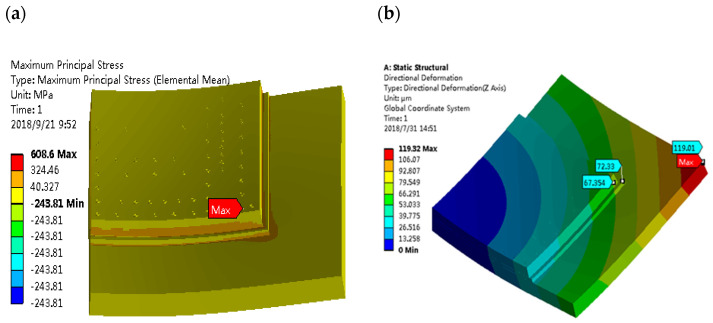
(**a**) Stress distribution in the package at 260 °C. (**b**) Warpage of the package at 260 °C.

The distribution of maximum principal stress in the global model is shown in Figure 7a. The maximum thermal stress is about 608.6 MPa, occurring at the corner of the chip which is the farthest point from the center of the chip, where the warpage in the Z direction (warpage) showed the largest value of 119.01 μm, shown in Figure 7b. It can be seen from the analysis of Figure 7 that the main cause of thermal stress occurs during the flip-chip solder reflow process. The high thermal stress induced by the CTE mismatch of the silicon chip with the substrate at the chip corner can be transmitted to the BEoL structure through the microbumps, and eventually causes fractures inside the low-*k* materials, or interfacial delamination in the BEoL stack. Therefore, a deeper investigation to the failure inside the BEoL stack is conducted based on the global modeling.

**Figure 8:**
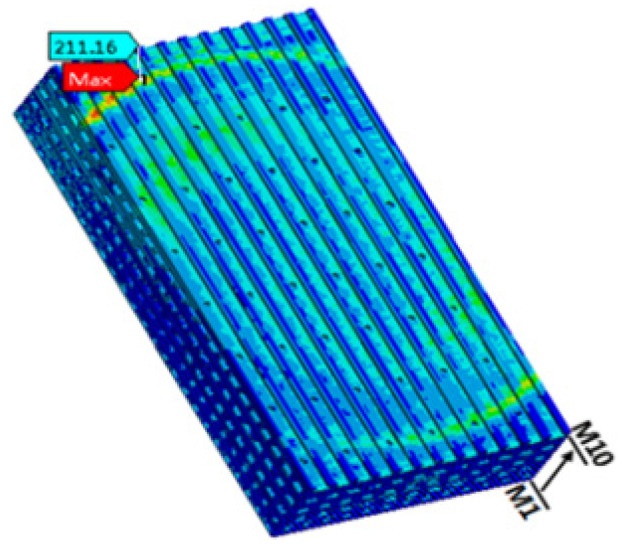
Maximum principal stress distribution in the BEoL structure at 260 °C.

It is expected that the first failure should occur in the BEoL stack immediately below the critical bump that is located farthest from the center of the chip. A local submodel for a much finer scale than the global model was established, as shown in Figure 2. Following the conventions of submodeling techniques, the displacement boundary of the submodel region in the global model is introduced to the local model by interpolation, serving as the displacement boundary conditions for the submodel. The distribution of maximum principal stress is shown in Figure 8, where it can be seen that the maximum stress of about 211.16 MPa is located in the low-*k* (M10) layer closest to the critical bump. This level of stress arising at the critical bump can cause delamination or cracking in the BEoL stack, leading to device failure.

## 3. Study on the Most Likely Failure Location of BEoL Stack

**Figure 9:**
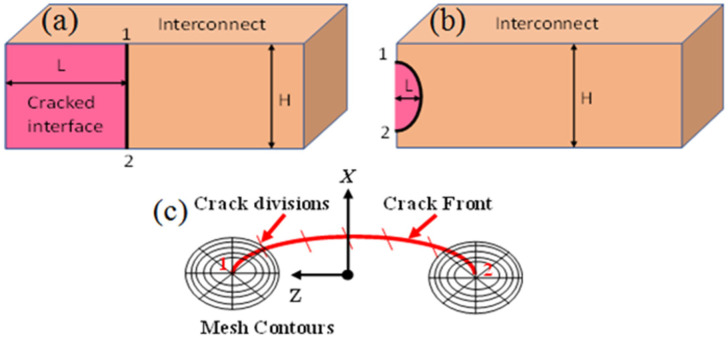
(**a**) A straight interfacial crack 1–2. (**b**) A semi-elliptical interfacial crack 1–2. (**c**) Mesh around the semi-elliptical crack front.

As described in the above sections, due to CTE mismatch between the silicon chip and the substrate, there is chip–package interaction which causes thermal stresses to be transmitted to the BEoL stack, with the largest stresses just below the critical bump. The BEoL stack consists of many interconnects and interfaces at many levels, and it is of interest to know where the most likely location of failure will be. This problem has been studied by some researchers using a fracture mechanics approach [13,19]. In this study, we will also use a fracture mechanics approach to solve this problem for the test vehicle studied. But unlike all the earlier researchers who used a straight crack front (Figure 9a), we use a more realistic elliptical crack at the interface between the barrier layer and the low-*k* dielectric, as shown in Figure 9b.

It is assumed that the major axis of the semi-elliptical interfacial crack is 0.30 μm and the minor axis 0.1 μm. A number of mesh contours were specified for meshing purposes as illustrated in Figure 9c. The optimum numbers of crack front divisions and mesh contours were set at six and five, respectively, for proper computational accuracy and efficiency.

### 3.1. Competitive Failure of Cracks along Different Interfaces

As cracks can occur at any location in the BEoL stack and appear at the interconnect–dielectric interfaces or inside the dielectric layers, when a thermal load is applied, such as during solder reflow, stress concentrations will develop at existing cracks which leads toa competition among all the cracks as to which will propagate first. The first failure will occur when the ERR exceeds the fracture toughness at one of the cracks. Hence, we performed finite element simulations in order to obtain the ERRs of cracks at various interfaces and locations in the BEoL in order to establish which cracks at which locations are most likely to propagate.

**Figure 10:**
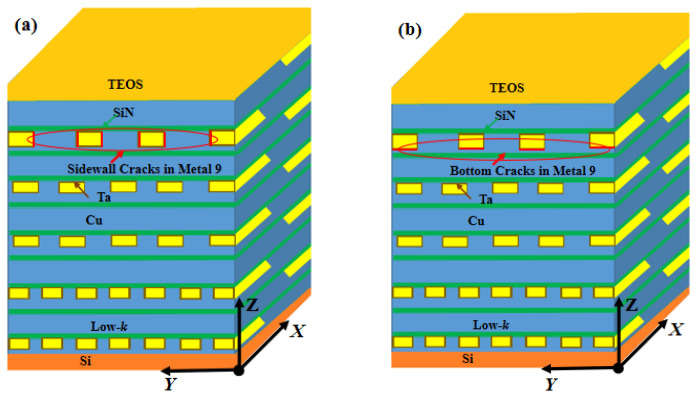
Cracks at (**a**) sidewalls and (**b**) bottom of M9 interconnects.

First, we considered the competitive crack propagation between cracks in the sides and bottoms of the interconnects in each metal layer. For this, interfacial cracks were first assumed to occur on the vertical sides of all the interconnect lines in a specified layer, and finite element simulations were performed in order to obtain the ERR. Figure 10a illustrates the case for M9. In this manner, the ERRs for sidewall cracks in each layer of the BEoL were calculated. Similarly, interfacial cracks were next assumed at the bottom of all the interconnects at each layer in turn, and the ERRs were calculated.

### 3.2. Variation of ERR along the Semi-Elliptical Crack Front

**Figure 11:**
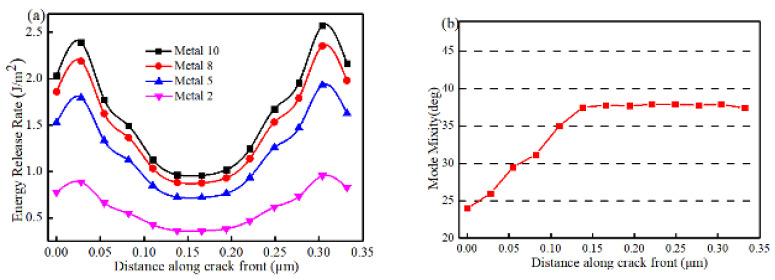
Variation of (**a**) ERR and (**b**) mode mixity along crack front.

The variation of ERR and mode mixity along the crack front 1–2 (Figure 9c) of a typical semi-elliptical crack at the bottom of the interconnects at different layers is shown in Figure 11. It can be seen that the distribution of ERR is approximately symmetric around the center of the crack front but slightly skewed towards the right because of the loading direction. The largest values of the ERR are not at the start of the crack front, (Point 1) nor at the end (Point 2), but are quite close to them. The lowest ERR occurs near the center of the crack front. From Figure 11b, it can be seen that the mode mixity is lowest at the beginning of the crack front (Point 1) and increases linearly along the crack front until about the middle of the crack. Thereafter it remains almost constant.

### 3.3. Variation of ERR with Metal Layer Number

**Figure 12:**
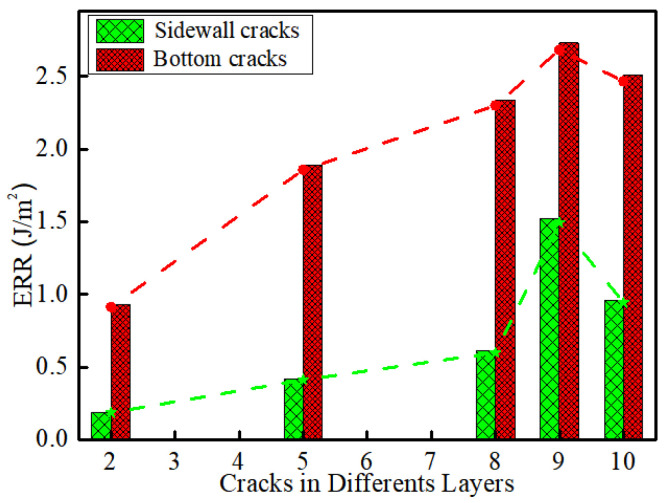
Variation of ERR with different metal layers.

The largest ERR of each of the side cracks and bottom cracks are plotted in Figure 12. It can be seen that, as the layer number increases, the ERR for both the sidewall cracks and the bottom cracks increases except for in the very last layer (M10), in which it decreases slightly. A similar trend was observed by Zhai et al. [19] for a flip chip with nine metal layers where M1–M7 had low-*k* ILD, while M8 and M9 had stiffer oxide ILD. They found that the largest ERR occurred at M6, with that at M7 slightly lower and those at M8 and M9 much lower as their ILD was an oxide. It can also be seen from Figure 12 that the ERR for the bottom crack at each layer is always greater than those for the sidewall cracks. Hence, for the chip studied here, layer M9 is most likely to fail first from a crack at the bottom of the interconnect.

### 3.4. Competitive Failure between Interfacial Cracks and Cracks in the Low-k ILD

**Figure 13:**
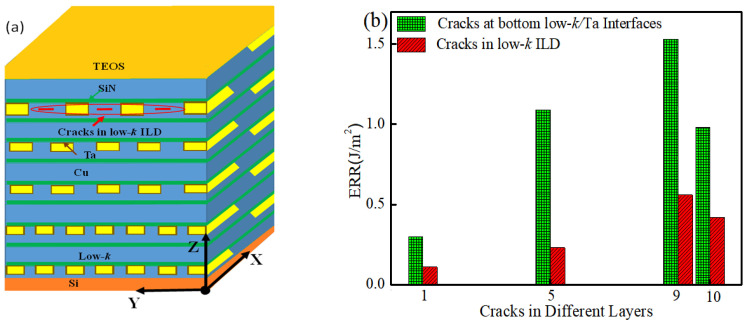
(**a**) Schematic diagram of cracks in low-*k* ILD layers at M9. (**b**) Comparison of ERR of interfacial cracks at interconnect sidewalls and in low-*k* ILD layers.

In addition to cracks at the Ta/low-*k* interfaces, there is also the possibility of cracking inside the low-*k* ILD as well. Here it was investigated by calculating the ERR of cracks located at the center of ILD between each two adjacent interconnects inside each layer. Figure 13a illustrates the model for the calculation of the M9 layer. The cracks are semi-elliptical and of the same size as mentioned before. Figure 13b shows the ERR of cracks in the interface of the sidewalls and in the low-*k* ILD for layers M1, M5, M9, and M10. The variations of the ERR inside the low-*k* ILD layers follow the same trend as those for the interfacial cracks, but its magnitude is significantly lower.

## 4. Experimental Study on Failure of BEoL Stack

### 4.1. Experimental Procedure

**Figure 14:**
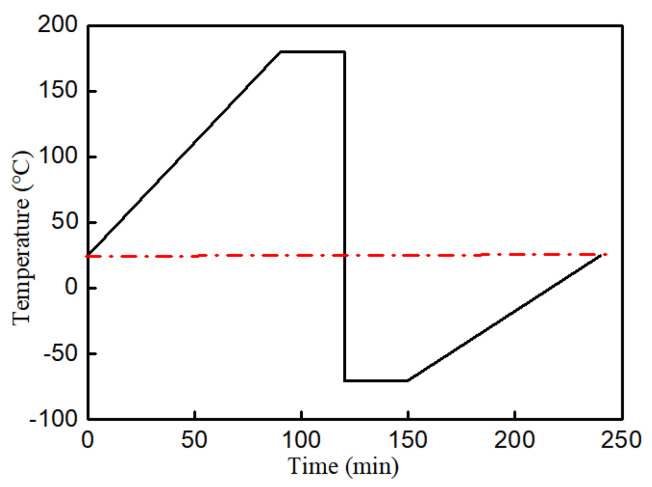
Time-temperature curve of the thermal shock test.

A thermal shock test, which induces thermal stress, was carried out in order to study the failure of the BEoL layer. A TSP-101-W-type thermal shock tester was used, which uses air as a medium for heating and cooling, and the whole cooling process from 180 °C to −70 °C can be completed in 10 s. A high-temperature target of 180 °C and a low-temperature target of −70 °C were held for 30 min, as shown in the temperature loading curve in Figure 14. The test lasted for 500 cycles. Ultrasonic scanning acoustic microscopy (C-SAM) with different probe frequencies (15 MHz/30 MHz/50 MHz/100 MHz/230 MHz) and different detection modes (dot scan/block scan/layer scan) were used to detect cracks, delamination, voids, and bubbles in the specimens after the thermal shock test.

### 4.2. Experimental Results

**Figure 15:**
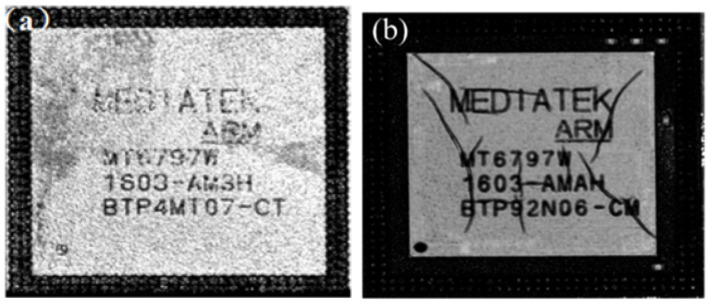
(**a**) No cracks on top surface of test vehicle after 500 thermal shock cycles. (**b**) Cracks inside the chip after 500 cycles of thermal shock.

After the thermal shock test, the package was scanned layer by layer using a high frequency (230 MHz) probe. As shown in Figure 15a, after 500 cycles of thermal shock, the top surface of the package remained intact and no large cracks were found. Scanning down to the chip layer revealed cracks in the silicon, as shown in Figure 15b. These cracks appear at the corners of the chip, and they are mostly radial.

**Figure 16:**
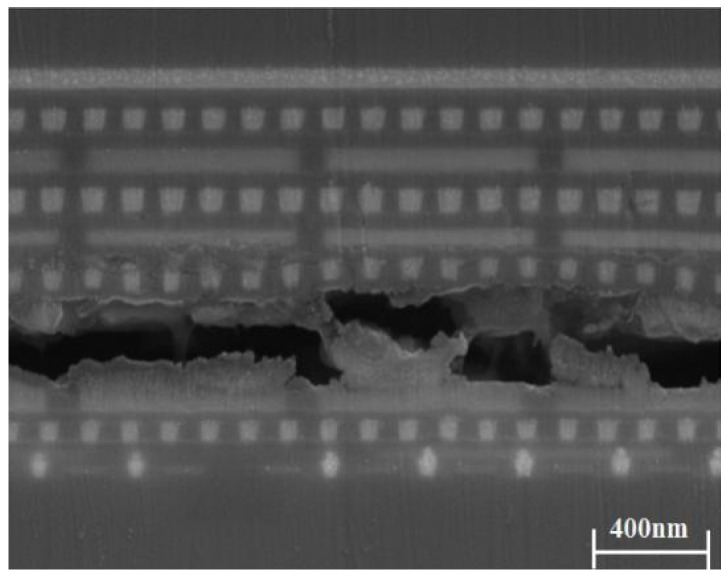
Cross-section of BEoL stack showing horizontal crack at M9.

FIB milling was used to obtain the detailed state of the BEoL stack after the thermal shock test. As shown in Figure 16, the M1–M8 layers in the BEoL stack had good integrity, but the M9 layer delaminated. The crack only appears in the horizontal plane at the bottom of the M9 interconnect, which corresponds to the highest layer location in the BEoL stack most likely to fail.

## 5. Conclusions

In this paper, the reliability issues of the IC chip with a feature size of 20 nm due to the chip–package interaction during the reflow process is studied. A representative volume element of the detailed complex BEoL structure is analyzed to obtain the equivalent mechanical properties of the BEoL stack. The result is then used in a finite element “global-local” two-level analysis to obtain the thermal stress distribution in the BEoL structure during solder reflow. A fracture mechanics approach using a novel semi-elliptical virtual crack is employed to calculate the ERR at various interfaces and locations in the BEoL stack which could then be used to predict the most likely mode and location of failure. The results show that:

(1) The microbumps furthest from the center of the chip have the greatest stress, and accordingly the largest PCB warpage.
(2) The crack ERR of the low-*k*/Ta interfaces are higher than that for cracks within the low-*k* material.
(3) Interfacial cracks in the M9 layer in the BEoL structure have the largest ERR among all layers and are hence more likely to propagate.
(4) The interfacial crack at the bottom of the interconnect is more likely to propagate than that at the sidewalls of the interconnect.
(5) The thermal shock experiments demonstrated that the dominant failure mode is the delamination in the second outer layer of the metal layer in the manner of Ta/low-*k* interfacial cracking at the bottom.

In summary, the numerical approach present in this paper can effectively handle the issue of modeling a complex IC package which stretches over incommensurable scales and provides well-validated predictions for the design of reliability for advanced Cu/low-*k* interconnects.

## Figures and Tables

**Table 1:** Dimensions of Components of the Package.

Component	Dimensions (h = Height, D = Diameter)
Si	6.8 mm × 6.8 mm × 150 μm
PCB	15 mm × 15 mm × 190 μm
SAC	D = 30 μm, h = 5 μm
Copper pillar	D = 30 μm, h = 20 μm
Cu Pad	D = 30 μm, h = 10 μm
BEoL layer	h = 8.2 μm
Al Pad	D = 20 μm, h = 3 μm
PI layer	D = 25 μm, h = 4 μm
Underfill	7 mm × 7 mm × 59 μm
passivation	h = 4 μm

**Table 2:** Mechanical Properties of the Package Materials.

Material	Elastic Moudulus(GPa)	Poisson’s Ratio	Themal Expansion Coefficient (ppm)
Cu	130	0.35	17.4
Ta	195	0.3	6.5
Low-*k*	10.6	0.3	15
Underfill	14.5	0.34	α_1_/α_2_:25/110; Tg = 135 °C
SiN	200	0.27	3
Passivation	211	0.27	3.2
Si	129	0.28	2.9
Al	74	0.33	21.5
PI	3.5	0.34	35
SAC	43	0.3	21.3 + 0.0175T (°C)

**Table 3:** Dimensions of the Different Components of the BEoL Stack.

Material	Pitch (nm)	Width (nm)	Thickness (nm)	Barrier Thickness (nm)	Etched Layer Thickness (nm)
Metal 1	200	800	400	30	40
Metal 2	200	800	400	30	40
Metal 3	200	800	400	30	40
Metal 4	300	850	500	40	45
Metal 5	300	850	500	40	45
Metal 6	300	850	500	40	45
Metal 7	500	900	600	50	60
Metal 8	500	900	600	50	60
Metal 9	500	900	600	50	60
Metal 10	700	1050	700	60	60
Si			750		

## Data Availability

The raw/processed data required to reproduce these findings cannot be shared at this time as the data also forms part of an ongoing study.

## References

[1] Zhang J., Zhang G., Gao Y., Sun R., Wong C.P. (2016). Ultra-low-κHFPDB-based periodic mesoporous organosilica film with high mechanical strength for interlayer dielectric. J. Mater. Sci..

[2] Xiao C., He H., Li J., Cao S., Zhu W. (2017). An effective and efficient numerical method for thermal management in 3D stacked integrated circuits. Appl. Therm. Eng..

[3] Li H., Tie J., Li J., Ye M., Zhang H., Zhang X., Pan Y., Wang Y., Quhe R., Pan F. (2017). High-performance sub-10-nm monolayer black phosphorene tunneling transistors. Nano Res..

[4] Fu S., Qian K., Ding S., Zhang W., Fan Z. Characterization of ultra-low k porous organosilica thin films. Proceedings of the IEEE International Conference on Solid-State and Integrated Circuit Technology.

[5] Li G., Zheng G., Ding Z., Shi L., Li J., Chen Z., Wang L., Tay A.A.O., Zhu W. (2018). High-performance ultra-low-k fluorine-doped nanoporousorganosilica films for inter-layer dielectric. J. Mater. Sci..

[6] Chai T.C., Zhang X., Li H.Y., Sekhar V.N., Hnin W.Y., Thew M.L., Navas O.K., Lau J., Mruthy R., Balakumar S. Impact of packaging design on reliability of large die Cu/low-k interconnect. Proceedings of the 2008 58th Electronic Components and Technology Conference.

[7] Bao A., Zhao L., Sun Y., Han M., Lee K. Challenges and opportunities of chip package interaction with fine pitch Cu pillar for 28 nm. Proceedings of the 2014 IEEE 64th Electronic Components and Technology Conference (ECTC).

[8] Jacob P. (2008). Surface ESD (ESDFOS) in assembly fab machineries as a functional and reliability risk—Failure analysis, tool diagnosis and on-site-remedies. Microelectron Reliab..

[9] Ryan V., Breuer D., Geisler H., Kioussis D., Karimanal K. CPI challenges to BEOL at 28 nm node and beyond. Proceedings of the IEEE International Reliability Physics Symposium.

[10] Lei M., Goldberg C., Kuo S.M. A simulation method for predicting packaging mechanical reliability with low/spl kappa/dielectrics. Proceedings of the IEEE 2002 International Interconnect Technology Conference (Cat. No.02EX519).

[11] Mercado L., Goldberg C., Kuo S.-M., Lee T.-Y., Pozder S., Yu T., Lee T. (2003). Analysis of flip-chip packaging challenges on copper/low-k interconnects. IEEE Trans. Device Mater. Reliab..

[12] Zhang X., Wang Y., Im J.H., Ho P.S. (2012). Chip–Package Interaction and Reliability Improvement by Structure Optimization for Ultralow-$k$ Interconnects in Flip-Chip Packages. IEEE Trans. Device Mater. Reliab..

[13] Liu X.H., Shaw T.M., Lane M.W., Liniger E.G., Questad D.L. Chip-Package-Interaction Modeling of Ultra Low-k/Copper Back End of Line. Proceedings of the IEEE International Interconnect Technology Conference.

[14] Wang G., Ho P.S., Groothuis S. (2005). Chip-packaging interaction: A critical concern for C/low k packaging. Microelectron. Reliab..

[15] Wang G. (2008). Thermal Deformation of Electronic Packages and Packaging Effect on Reliability for Copper/Low-k Interconnect Structures.

[16] Wang G., Groothuis S., Merrill C., Ho P.S. Investigation of interfacial delamination for Cu/low k structures during flip-chip packaging. Proceedings of the Conference on Thermal & Thermomechanical Phenomena in Electronic Systems.

[17] Gao S., Smith R.S., Cho J.K., Choi S., Kannan S., Chua E., Geisler H., Kuechenmeister F. (2014). Chip Packaging Interaction (CPI) with Cu Pillar Flip Chip for 20 nm Silicon Technology and Beyond. ECS J. Solid. State Sci. Technol..

[18] Uchibori C.J., Ho P.S., Nakamura T. Chip Package Interaction and Mechanical Reliability Impact on Cu/ultra low-k Interconnects in Flip Chip Package. Proceedings of the International Conference on Solid-state & Integrated-Circuit Technology.

[19] Tambat A., Lin H.Y., Subbarayan G., Jung D.Y., Sammakia B. (2012). Simulations of Damage, Crack Initiation, and Propagation in Interlayer Dielectric Structures: Understanding Assembly-Induced Fracture in Dies. IEEE Trans. Device Mater. Reliab..

